# Regulation of Caspase-8 Activity at the Crossroads of Pro-Inflammation and Anti-Inflammation

**DOI:** 10.3390/ijms22073318

**Published:** 2021-03-24

**Authors:** Jun-Hyuk Han, Jooho Park, Tae-Bong Kang, Kwang-Ho Lee

**Affiliations:** 1 Department of Applied Life Sciences, Graduate School, BK21 Program, Konkuk University, Chungju 27478, Korea; digit0516@naver.com (J.-H.H.); kjhdn@kku.ac.kr (J.P.); kwangho@kku.ac.kr (K.-H.L.); 2 Department of Biomedical Chemistry, College of Biomedical & Health Science, Konkuk University, Chungju 27487, Korea; 3 Department of Biotechnology, College of Biomedical & Health Science, Konkuk University, Chungju 27487, Korea

**Keywords:** caspase-8, inflammasome, inflammation, necroptosis, pyroptosis, apoptosis

## Abstract

Caspase-8 has been classified as an apoptotic caspase, and its initial definition was an initiator of extrinsic cell death. During the past decade, the concept of caspase-8 functioning has been changed by findings of its additional roles in diverse biological processes. Although caspase-8 was not originally thought to be involved in the inflammation process, many recent works have determined that caspase-8 plays an important role in the regulatory functions of inflammatory processes. In this review, we describe the recent advances in knowledge regarding the manner in which caspase-8 modulates the inflammatory responses concerning inflammasome activation, cell death, and cytokine induction.

## 1. Introduction

Mammalian caspases have classically been divided into inflammatory and apoptotic caspases based on their cellular functions. Inflammatory caspases, such as caspase-1, -4, -5, and -11, induce inflammation by engaging in the activation of the inflammasome and an inflammatory form of programmed death, called pyroptosis [1]. In contrast, apoptotic caspases take part in an immune silencing form of programmed cell death and include the initiator caspases, caspase-2, -8, -9, and -10, and the effector caspases, caspase-3, -6, and -7 [1].

**Figure 1:**
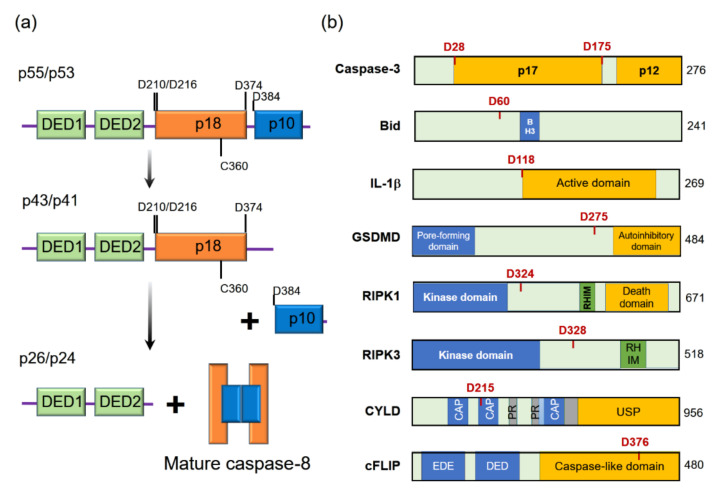
(**a**) Schematic showing the important residues involved in activation of human caspase-8. Procaspase-8 (p55/p53) is an inactive precursor composed of two death effector domains (DEDs), a large subunit (p18) and a small subunit (p10). The maturation of caspase-8 is derived by its auto-processing between p18 and p10 and subsequently between p18 and the pro-domain [3,4]. The mature caspase-8 consists of two large subunits and two small subunits. (**b**) Substrates of caspase-8 and targeted cleavage sites. Caspase-8 is involved in inflammation processes in a variety of ways: immune silent apoptosis induction through the cleavage of caspase-3 and bid [6,7,8]; inflammation induction by processing IL-1β and gasdermin D (GSDMD) [9,10]; inhibition of inflammatory cell death (necroptosis) by targeting Receptor-interacting protein kinase (RIPK)1, RIPK3 and cylindromatosis (CYLD) [11,12,13,14]; NF-κB activation through cellular Fas-associated protein with death domain (FADD)-like IL-1β-converting enzyme (FLICE)-inhibitory protein (cFLIP) cleavage fragments [15].

Among these, caspase-8 is particularly interesting in terms of multiple functions in a variety of inflammatory processes. Caspase-8 was found to be an apical enzyme that initiates the extrinsic apoptotic cell death pathway induced by the activation of death receptors, such as tumor necrosis factor receptor (TNFR)1, Fas cell surface death receptor (FAS), and death receptors (DRs) [2]. Caspase-8 is synthesized as a zymogen, which is an inactive precursor containing two N-terminal death effector domains (DEDs) and a C-terminal protease domain containing a large subunit (p18) and a small subunit (p10). The activation of caspase-8 is derived by its dimerization and autoprocessing by the proximity-driven mechanism [3,4], and the active caspase-8 can cleave effector caspases and other substrates (Figure 1a) [5].

Although earlier studies on caspase-8 have focused on its role in the cell death pathway, recent investigations have discovered additional functions of caspase-8 for the regulation of inflammation in various means depending on its substrates (Figure 1b). In this review, we describe the recent updates of the role of caspase-8 in the regulation of inflammation, e.g., inflammasome activation, IL-1β processing, and cytokine induction.

## 2. Anti-Inflammatory Functions of Caspase-8

Classically, caspase-8 is believed to have an anti-inflammatory function because of its role in apoptosis; however, several recent studies have revealed that caspase-8 also serves an anti-inflammatory function in several ways.

### 2.1. Induction of Apoptosis

Apoptosis is generally considered a type of cell death that is immunologically silent because the cells undergoing apoptosis are quickly eliminated by local phagocytes. In mammalian cells, apoptosis has been subdivided into intrinsic and extrinsic apoptosis [16]. Intrinsic apoptosis is triggered by the signal originating from intracellular organelles, such as DNA damage.

Extrinsic apoptosis is initiated by the binding of death ligands, including Fas ligand (FasL), tumor necrosis factor (TNF), and TNF-related apoptosis-inducing ligand (TRAIL) to their corresponding death receptors, such as Fas, TNFRI, death receptor (DR)3, DR4, and DR5. Caspase-8 is crucial in inducing cell death in this pathway [17,18,19,20]. Upon the activation of death receptors, a death domain (DD)-containing adaptor protein, Fas-associated protein with death domain (FADD) binds at the cytoplasmic domain of receptors through homotypic DD association to form a death-inducing signaling complex (DISC). In the case of TNF-induced apoptosis, FADD does not directly bind to the TNFRI complex (complex I) but rather binds to a cytosolic complex (complex II) lacking the TNFRI [16].

The conformational change of FADD by the binding to the complexes allows DED-containing proteins, such as procaspase-8 or FLIP_L,_ to bind to complex-associated FADD through the interaction of DED, which is crucial for the activation of caspase-8 and the subsequent induction of apoptosis [16].

The recruitment of procaspase-8 and FLIP_L_ to the complex leads to the formation of DED filaments through the repeated self-assembly by different types of DED integrations, the so-called type I, II, III interactions [21,22,23,24]. At these filaments, procaspase-8 is activated by the homo-oligomerization of two procaspase-8 or the hetero-oligomerization of procaspase-8 and FLIP_L_ [25,26,27,28]. The catalytic activity of procaspase-8 gained by the homo-oligomerization may allow their autocatalytic processing at two sites, between a large subunit and a small subunit, and subsequently between DED2 and a large subunit [21,29].

**Figure 2:**
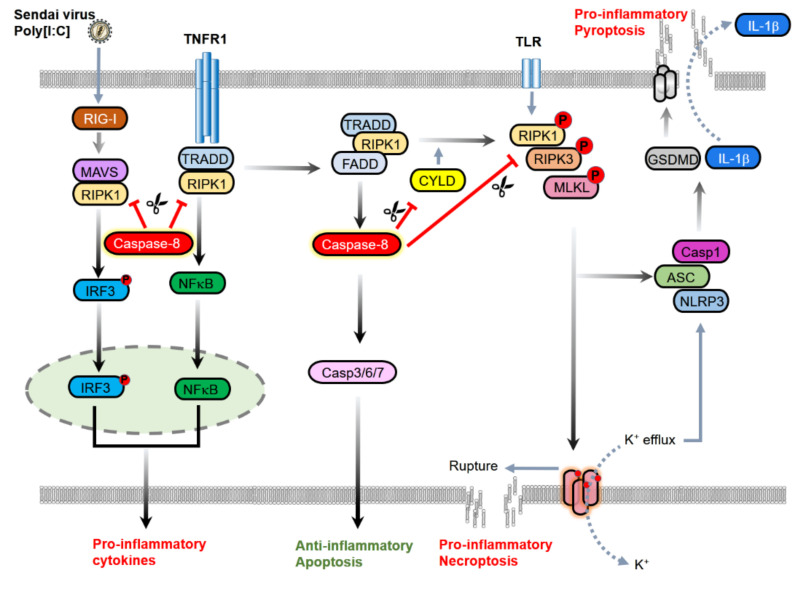
Caspase-8 contributes to the inhibition of inflammation through the induction of apoptosis, inhibition of necroptosis, and limiting of proinflammatory processes. Upon stimulation of death receptors, such as TNFR1, caspase-8 forms a complex with FADD, RIPK1, and Tumor necrosis factor receptor type 1-associated DEATH domain protein (TRADD) and is activated. The activated caspase-8 induces apoptotic cell death through the processing of effector caspases, such as caspase-3, -6, and -7. Caspase-8 inhibits necroptosis, a cell death mode that leads to inflammation by targeting the pro-necroptotic molecules RIPK1, RIPK3, and CYLD. In addition, caspase-8 is required for the inhibition of Toll-like receptor (TLR)-induced, MLKL-mediated inflammasome activation. The caspase-8 cleavage of RIPK1 also contributes to limiting proinflammatory cytokine production mediated by NF-κB or IRF3 in response to TNF or Sendai virus. Blue arrows indicate activation and red blunt arrows represent inbibition. The blue dot arrows indicate the release of molecules.

The processed caspase-8, then, is dissociated from the complex and activates downstream executioner caspases, such as caspase-3, -6, and -7, by cleaving them, ultimately leading to cell death (Figure 2).

This apoptotic process is important not only for development but also for eliminating potentially dangerous cells, such as inflamed, infected, and damaged cells [2], which eventually regulates inflammation and maintains body homeostasis.

### 2.2. The Inhibition of Necroptosis

Traditionally, in contrast to apoptosis, necrosis has been considered as an uncontrolled process of cell death, where cells died accidentally as a result of environmental insults [30].

A decade ago, a controlled type of necrosis, termed necroptosis, was identified via the discovery of its regulatory molecule RIPK3 [31,32], and this finding provided new insight into the novel role of caspase-8. Many subsequent studies showed that necroptosis occurs under inhibition of the apoptotic signal, including lack of caspase-8 function, and concluded that caspase-8 is able to block necroptosis [13,28,33,34].

Necroptosis leads to cell swelling, plasma membrane rupture, and the release of intracellular molecules normally contained by the cell membrane, which stimulates the surrounding immune cells to induce inflammation [35]. Necroptosis can be activated by a variety of stimuli, such as extracellular and intracellular ligands of the death receptor family [36]. The initial sign of the role of caspase-8 in necroptosis was found in the analysis of tissue-specific caspase-8 knockout mice [37]. The hematopoietic stem cells showed impaired differentiation to macrophages and increased cell death in the absence of caspase-8 [37]. At that time, however, it was not realized that the lack of caspase-8 facilitates another type of programmed cell death. Subsequently, the finding of RIPK3 shed light on how caspase-independent cell death occurs upon stimulation by TNFR [31,32]. The activation of TNFR recruits downstream molecules, RIPK1, RIPK3, and MLKL, to form a complex called necrosome in the absence of caspase-8 or in the lack of its activity (Figure 2) [38,39,40].

The means of inhibiting necroptosis via caspase-8 is through the cleavage of its substrates, such as RIPK1, RIPK3, and CYLD [11,12,13]. Interestingly, heterodimerization of caspase-8 with its substrate cFLIP_L_ is required for the cleaving and inactivating of proteins that promote necroptosis [28]. Caspase-8 cleaves RIPK1 at Asp325 (or Asp324 in humans) [11,41] and RIPK3 at Asp323 (Asp328 in human) [12] to prevent the interaction of two kinases, ultimately inhibiting necroptosis (Figure 1b) [11,41]. Heterozygous non-cleavable ripk1 D325A (±) cells and mice are more susceptible to TNF-induced cell death than wild-type cells or mice [41]. This indicates that caspase-8 is essential for limiting aberrant cell death in response to TNF through the cleavage of RIPK1 [41]. A deubiquitylating enzyme, CYLD, drives necroptosis by removing the ubiquitin chain from RIPK1 to facilitate its interaction with RIPK3 [13], implying the restriction of CYLD function by caspase-8 is required by its enzymatic activity (Figure 1b) [13].

Therefore, many transgenic mice that had a tissue-specific deletion of the *casp8* gene showed spontaneous inflammation [42,43,44], and mice lacking caspase-8 or C362A died in a necroptosis-dependent manner during embryogenesis [28,33,41,45]. In line with these results, caspase-8 deficiency in humans is associated with the early onset of inflammatory bowel disease [46].

### 2.3. Inhibition of Inflammasomes

Inflammasomes are characterized by the formation of a multiprotein complex composed of caspase-1, and the adaptor protein, ASC, and upstream sensors, such as NOD-like receptors (NLRs), pyrin, and AIM2. The consequence of their activation is the release of proinflammatory cytokines interleukine-1β and -18, and induction of pyroptosis, a type of programmed cell death [47,48,49]. Although pyroptosis was long regarded as caspase-1-mediated cell death, the finding of gasdermin D (GSDMD), a substrate of active caspase-1 and caspase-11/4/5, redefined the concept of pyroptosis as GSDMD-mediated programmed necrosis. As a result that the GSDMDs cleaved by caspases form plasma membrane pores, cells undergo death with osmotic lysis and the release of inflammatory intracellular contents [50,51,52]. Therefore, misregulation of inflammasome activation is implicated in various inflammatory diseases [53].

The first report related to the inhibitory role of caspase-8 on inflammasome activation was shown in the analysis of dendritic cells from CD11C-Cre/*casp8* floxed mice [54]. In the deficiency of caspase-8 in bone-marrow-derived dendritic cells (BMDCs), LPS-primed cells evoke the NLRP3 inflammasome activation spontaneously without a second stimulus [54]. Mechanistically, knockdown of *ripk1*, *ripk3*, and *mlkl* blunted the IL-1β secretion, indicating their involvement in inflammasome activation in this context. In line with these findings, the lack of caspase-8 results in IL-1β secretion in the absence of ATP without undergoing necroptosis and IL-1β secretion is inhibited by the addition of necrostatin-1, a RIPK1 kinase inhibitor [55]. Additionally, in the presence of a caspase inhibitor, dsRNA induced ASC speck formation, inflammasome activation, and IL-1β release from macrophages without the second stimulus in an MLKL-dependent manner [56].

Overall, these results suggest that TLR signaling can induce NLRP3 inflammasome activation through the necroptotic machinery proteins, but in a cell death-independent manner, and such activation might be regulated by caspase-8. It remains to be determined how exactly caspase-8 inhibits RIPK1, RIPK3, and MLKL-associated inflammasome activation. A plausible explanation arises from a study that showed that MLKL activation induces the process and release of IL-1β through the triggering of potassium efflux in THP1 cells [57]. In addition, this study proved that MLKL activation is sufficient to induce NLRP3-mediated IL-1β release without GSDMD processing, which indicates MLKL can be an endogenous activator of inflammasome [57] in a cell death-independent manner.

In addition, recent results from the analysis of mice (*Casp8^c362s/c362s^*) expressing enzymatically inactive caspase-8 supported the notion that caspase-8 functions as a negative regulator of inflammasome activation [58]. As expected, like a caspase-8 knockout, the expression of enzymatically inactive caspase-8 (C362S) leads to embryonic lethal in mice by induction of necroptosis and inhibition of necroptosis by additional ablation of *mlkl* gene rescued embryonic death and the cardiovascular phenotype of *Casp8^c362s/c362s^* mice [58]. These mice, however, showed premature death with severe intestinal inflammation with the formation of ASC speck and caspase-1 activation, indicating necroptosis-independent inflammation. Interestingly, such necroptosis-independent phenotypes were prevented by either caspase-1 or ASC depletion, which implies that the activation of inflammasome promotes caspase-8 (C362S)-mediated tissue pathology under blocking of necroptosis, and the catalytic activity of caspase-8 is required for the suppression of inflammasome activation in epithelial cells in certain cellular contexts [58]. The result showing the inflammasome activation found in *Casp8c^362s/c362s^ mlkl^−/−^* does not appear in *Casp8^−/−^ mlkl^−/−^* mice indicates that the scaffolding function of caspase-8 is also required for inflammasome activation in caspase-8 (C362S) mice. Therefore, further studies are needed to understand how catalytically dead caspase-8 exactly induces ASC speck formation and caspase-1 activation and which molecules could be a substrate for the catalytic activity of caspase-8.

### 2.4. Inhibition of Inflammatory Signaling Complex

A series of results from the analysis of genetically modified caspase-8 mice revealed alternative roles of caspase-8 for the inhibition of inflammatory processes [42,59,60,61].

In response to M-CSF, transient the nuclear factor kappa light chain enhancer of activated B cells (NF-κB) activation occurs in macrophages during differentiation, and the sustained NF-κB activation is prevented by the C-terminal fragment of RIPK1 cleaved by caspase-8 [59]. The depletion of caspase-8 in cultured keratinocytes significantly enhances inflammatory cytokine production in response to DNA transfection, indicating caspase-8 can suppress an inflammatory pathway downstream of the cytosolic innate DNA receptor [42].

In addition, caspase-8 suppresses cytosolic RNA sensor retinoic acid-inducible gene I (RIG-I)-induced proinflammatory gene expression through cleavage of RIPK1 in murine keratinocytes, which is recruited to a complex of RIG-I and its adapter molecule MAVs [60]. The deficiency of caspase-8 in dendritic cells results in a systemic autoimmune disease in mice, and the cells show uncontrolled TLR activation characterized by massive cytokine induction, such as IL-12, IL-6, and TNF-α, in a RIPK1-dependent manner [55].

More recently, *Casp8^−/−^ rip3^−/−^* mouse embryo showed marked upregulation of several inflammatory genes compared to *rip3^−/−^* mouse embryo, suggesting the deficiency of caspase-8 can facilitate the activation of proinflammatory genes in a necroptosis-independent manner [61]. Although the upregulation of inflammatory gene expression in *Casp8^−/−^ rip3^−/−^* mouse embryo is attenuated by additional deletion of the *ripk1* gene, the underlying mechanism by which caspase-8 regulates RIPK1 to suppress inflammatory gene expression is not yet understood.

In addition, recent two studies also demonstrate the importance of caspase-8-mediated RIPK1 cleavage in maintaining inflammatory homeostasis [62,63]. The studies showed the expression of non-cleavable RIPK in humans leads to RIPK1-dependent overexpression for inflammatory cytokines and to an autoinflammatory syndrome [62,63].

## 3. Proinflammatory Functions of Caspase-8

**Figure 3:**
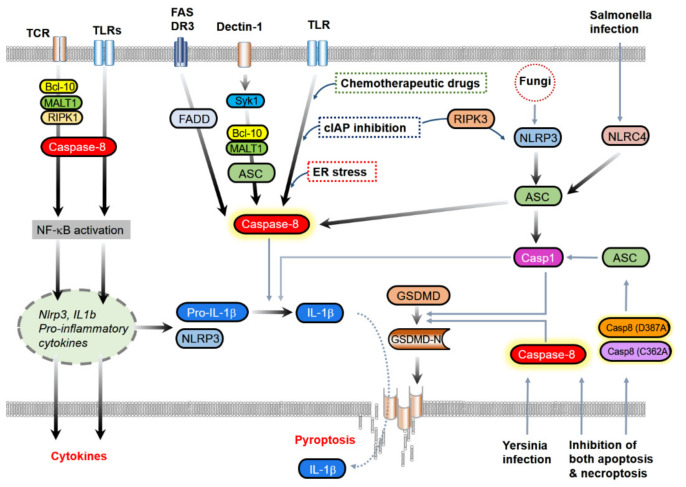
Caspase-8 promotes inflammation through IL-1β and GSDMD processing, activation of the inflammasome, and NF-κB activation. Caspase-8 can directly cleave IL-1β upon stimulation of FAS/Dr3, Dectin-1, and TLR with cellular stress conditions, such as chemotherapeutic drugs, ER stress, and cIAP inhibition. Caspase-8 mediates pyroptosis by direct processing of GSDMD in response to *Yersinia* infection or by induction of inflammasome activation through caspase-1 activation by its scaffolding function. Fas cell surface death receptor (FAS) and death receptor 3 (DR3) signal FADD-caspase-8 to process IL-1β and dectin-1 ligation induces IL-1β processing through caspase-8 assisted by Bcl-10, MALT-1, and ASC. Caspase-8 contributes to T-cell receptor (TCR)- or TLR-induced NF-κB activation, production of proinflammatory cytokines, and priming of the inflammasome.

Although caspase-8 appears to strongly inhibit inflammation as described above, it paradoxically contributes to inflammation, including cytokine production, NF-κB activation, and IL-1β processing, in an inflammasome activation-dependent and -independent manner (Figure 3).

### 3.1. Contribution to NF-κB Activation and Cytokine Induction

The initial studies on the relationship between caspase-8 and NF-κB showed that the overexpression of caspase-8 in HEK293T cells induced NF-κB activation, and this NF-κB activation was not dependent on its protease domain but rather on the DED-containing pro-domain [64,65]. Later, several studies proposed that caspase-8-mediated NF-κB activation is required for proper lymphocyte activation in humans [66,67]. Humans with an inherited deficiency of caspase-8 have shown immunodeficiency with defects in the activation of lymphocytes, including T, B, and NK cells [68]. Human peripheral blood leukocytes treated with zVAD or caspase-8-deficient human lymphocytes showed impaired NF-κB activation upon stimulation of the antigen receptor or Toll-like receptor 4 [66].

In line with these findings, the depletion of caspase-8 in mouse B cells resulted in impaired TLR4-induced B cell proliferation because of impaired NF-κB activation [69]. In addition, NLRP3 expression by TLR3 stimulation is mediated by the caspase-8 scaffolding function [56].

Caspase-8 is also required in the death receptor ligation-induced NF-κB signaling pathway [70,71,72,73]. TNF-related apoptosis-inducing ligand (TRAIL) is known to induce NF-κB dependent proinflammatory cytokine and chemokine expression. Knockdown or depletion of caspase-8 suppressed both TRAIL-mediated apoptosis and cytokine production, while the inhibition of caspase-8 enzymatic activity did not block cytokine production, indicating the scaffolding role of caspase-8 is required for TRAIL-induced cytokine induction [70,71,72]. Fas ligand (CD95L)-induced NF-κB activation also requires caspase-8, particularly its pro-domain [73].

In some experiment settings, however, the enzymatic activity of caspase-8 is necessary for NF-κB activation and cytokine production in mice [74]. It was shown that caspase-8, and its catalytic activity, is required for ASC-mediated NF-κB activation and IL-8 production [75]. Consistent with this, caspase-8 is required for optimal production of inflammatory cytokine (TNF, IL-6, IL-12) upon *Yersinia* infection in both macrophages and in mice. For this, enzymatic activity, but not self-cleavage of caspase-8, was required [74].

In addition, macrophages from *ripk3/casp8* double knockout mice or *mlkl/casp8* double knockout mice showed less cytokine induction upon *Yersinia* infection and TLR stimuli, such as LPS, Pam3CSK, CpG, and polyinosinic:polycytidylic acid (Poly[IC]) [76,77]. Moreover, the pharmacological inhibition of caspase-8 also suppressed LPS-induced cytokine expression (TNF, IL-1β, MIP-1, and MCP-1 mRNA) and inflammasome components in raw 264.7 cells and blunted inflammation-induced angiogenesis [78].

The contribution of caspase-8 in cytokine induction was shown even in the pathogenesis of malaria [79]. In malaria models in mice, caspase-8 controls the expression of pro-IL-1β and TNF-α as well as their release in plasma [79].

The means by which caspase-8 mediates NF-κB activation vary depending on cell type [66,68,69,80].

In human T cells, caspase-8 serves as a link for the CARMA1-Bcl10-MALT1 (mucosa-associated lymphatic tissue) (CBM) and IKK complex to induce NF-κB activation [66], and an inherited genetic deficiency of caspase-8 in human leads to immunodeficiency by impaired activation of T, B, and NK cells [68]. Caspase-8 also associates with the IKK complex in B cells to help NF-κB nuclear translocation and its transcriptional activity [69], and it is also necessary for optimal nuclear translocation of an NF-κB family member c-Rel [81]. In the TRAIL-induced cytokine induction, procaspase-8 is required for the recruitment of RIPK1 to the TRAIL receptor complex to promote NF-ΚB activation [70].

Caspase-8 can contribute to NF-κB activation indirectly by cleaving its paralogue cFLIP_L_ [80,82]. In primary T and B cells, the caspase-8-cleaved fragment of cFLIP_L_, the p43 fragment, was shown to mediate NF-κB activation in T cells through interaction with the TRAF2, RIP1, and IKK complex [82,83].

In addition to its role in NF-κB activation, caspase-8 promotes cytokine production in response to TLR3 and TLR4 stimulation through the inactivation of NEDD4-binding protein that is known as a suppressor of cytokine production [84], which may explain why the cytokine production was impaired in caspase-8 deficient macrophages upon stimulation of TLR3 and TLR4 [85].

### 3.2. IL-1β Processing

Interleukin-1β (IL-1β) is a potent proinflammatory cytokine and is primarily produced from monocytes, macrophages, dendritic cells, neutrophils, and natural killer (NK) cells in response to microbial ligands and cytokines, such as TNF and IL-1β [86].

IL-1β is produced as an inactive precursor form in cells, and its processing and secretion are mediated by the action of caspase-1, which is activated via recruitment to a multiprotein complex, inflammasome [47,48,49]. Although IL-1β is essential for host defense against pathogen and injury [86], it is a major pathogenic mediator of autoimmune, degenerative, and various inflammatory diseases [53].

Until recently, caspase-8 has not been studied for its potential to process pro-IL-1β; however, recent evidence has indicated that caspase-8 is involved in the cleavage of pro-IL-1β by distinct mechanisms, as described below.

#### 3.2.1. The Association with Classical Inflammasome Components

Depending on the cellular context, caspase-8 can be integrated into the inflammasome complex and participates in inflammasome activation and be activated by NLRP3, AIM2, and NLRC4 inflammasome [77,87,88]. During *Salmonella* infection in macrophages, caspase-8 was shown to assemble the inflammasome complex through ASC and NLRC4. The recruited caspase-8 is activated by auto-proteolysis in the complex and contributes to IL-1β processing directly [77].

In addition, caspase-8 also participates in the classical NLRP3 inflammasome activation induced by LPS and nigericin in dendritic cells. In particular, in the absence of caspase-1/11, caspase-8 is engaged in NLRP3 inflammasome and is activated to cleave pro-IL-1β [88]. Similarly, an internalized fungal pathogen, *Cryptococcus* neoformans, led to the activation of noncanonical NLRP3-ASC-caspase-8 in dendritic cells in the absence of caspase-1 [89].

In addition, caspase-8 is also activated in the AIM2 inflammasome in macrophages induced by cytosolic DNA but does not seem to be involved in the processing of IL-1β [87]. The integration of caspase-8 into inflammasomes is mediated by ASC [77,87,88], in which the DED-domain of caspase-8 can interact with the PYD domain of ASC [90].

Caspase-8 is able to regulate NLRP3 inflammasome activation upstream of NLRP3 under certain conditions [56,91]. The caspase-8/FADD complex is required for cytoplasmic dsRNA-induced NLRP3 activation in macrophages [56]. In the same context, the absence of inhibitors of apoptosis proteins (IAPs) promotes RIPK3-mediated caspase-8 activation in response to LPS, which leads to NLRP3 inflammasome activation and pro-IL-1β processing [91].

Furthermore, caspase-8 contributes to inflammasome activation by mediating the priming step, such as the expression of NLRP3 and pro-IL-1β [76,85,92]. This function might be caused by the engagement of caspase-8 with NF-κB activation.

More recently, it was shown that non-cleavable caspase-8 (D387A) expression in mice under depletion of both FADD and MLKL could induce caspase-1 activation through the induction of ASC oligomerization [93].

#### 3.2.2. Direct Processing of IL-1β

In addition to its role in the modulation of the inflammasome complex and IL-1β mRNA expression, caspase-8 can directly cleave pro-IL-1β in certain cellular contexts.

In the first report on the role of caspase-8 in IL-1β processing [9], macrophages induced the cleavage of pro-IL-1β in response to TLR 3 and 4 stimulation by poly[I:C] and LPS under inhibition of protein synthesis in a caspase-1 independent manner. In contrast, the inhibition of caspase-8 activity by peptide inhibitors, or knockdown of caspase-8, inhibits the processing, suggesting a novel role for caspase-8 in pro-IL-1β processing. In the same study, it was proved that recombinant caspase-8 could cleave pro-IL-1β at the same site as caspase-1.

Later, several studies further confirmed caspase-8 cleavage of IL-1β that is independent of caspase-1 and -11 in certain contexts. Dectin-1 is a pattern recognition receptor that recognizes β-glucans from fungal pathogens. However, the CARD9-Bcl-10-MALT1 scaffold is required for the expression of pro-IL-1β in dendritic cells upon dectin-1 ligation, the recruitment of caspase-8 and ASC into this scaffold induces the pro-IL-1β processing of caspase-8 [94].

However, subsequently, in similar experiments, slightly different results were shown. In mouse dendritic cells, caspase-8 plays a role in IL-1β processing by promoting NLRP3 inflammasome activation in response to β-glucans stimulation and caspase-8 was also found for NLRP3-dependent IL-1β cleavage in response to heat-killed *C. albicans* [95]. These findings indicate the bioactive IL-1β secretion in response to fungal components is induced by NLRP3 inflammasome activation and caspase-8 also promotes IL-1β processing.

In addition, caspase-8 is also required for death receptor or TLR-induced IL-1β processing. The activation of death receptor 3 (DR3) induces the caspase-8-mediated IL-1β processing and secretion in monocyte-derived macrophages and human intestinal myeloid cells [96]. Similarly, the ligation of the Fas ligand activates caspase-8 in TLR-primed macrophages or dendritic cells, which induces the processing of IL-1β and IL-18 in an inflammasome-independent manner [97]. During experimental autoimmune encephalomyelitis (EAE) pathogenesis, LPS-induced IL-1β processing in microglia is mediated by caspase-8 through the formation of Interleukin-1 receptor-associated kinase (IRAKM)-caspase-8-ASC complex [98].

In dendritic cells, TLR4 ligation promotes the assembly of a complex that contains RIPK1, RIPK3, and FADD, and the caspase-8 activation occurs in the complex for IL-1β processing [99,100]. In this process, RIPK3 works as a positive regulator of caspase-8 activity that induces pro-IL-1β processing from LPS-stimulated BMDCs [99]. Histone deacetylase (HDAC) inhibitors are known to promote caspase-8-mediated IL-1β processing without the second stimulus in response to LPS stimulation in both dendritic cells and macrophages [100]. Co-stimulation of BMDCs with LPS and chemotherapeutic drugs promotes caspase-8-mediated pro-IL-1β processing and release. Proapoptotic agents, such as doxorubicin or staurosporine, induce IL-1β release from LPS-stimulated BMDCs in the absence of a second stimulus. Notably, the release of IL-1β is not affected by depletion of caspase-1 and -11 or inhibition of caspase-1 but is significantly suppressed in caspase-8 depleted BMDC cells [92]. These data suggest that TLR4 induces the assembly of caspase-8-based signaling complexes that are able to process IL-1β in response to chemotherapeutic drugs [92].

The TLR4 stimulation in bone-marrow-derived macrophages (BMDMs) undergoing endoplasmic reticulum (ER) stress also showed caspase-8 cleavage of IL-1β [101]. Cells treated with ER-stress-inducing drugs, tunicamycin or thapsigargin, are able to drive the release and processing of pro-IL-1β in response to LPS stimulation, and the processing of pro-IL-1β can normally occur even in the absence of ASC, indicating the classical inflammasome activation is dispensable. In contrast, the processing of pro-IL-1β was abolished by depletion of caspase-8 [101].

Similarly, TLR4 stimulation under cIAP inhibition triggers cleavage of IL-1β both by the NLRP3-caspase-1 and caspase-8 in a caspase-1-independent manner [102].

Overall, these data indicate that TLR4 stimulation in the context of ER stress, cIAP inactivation, or chemotherapeutic drugs drives IL-1β processing in a caspase-8-dependent manner rather than classical inflammasome.

### 3.3. Induction of Pyroptosis

Pyroptosis is mediated by the N-terminal fragment of GSDMD, and the processing of GSDMD is known to be governed by activated caspase-1 or -11; however, recent studies suggest there is an additional pathway regulating GSDMD processing by caspase-8 [103,104].

*Yersinia* infection is known to induce macrophage death via inhibition of cell survival pathways, such as NF-κB and MAPK signaling [105,106,107]. Mechanistically, the inhibition of TAK1 by a *Yersinia* outer protein, YopJ, induces caspase-8-mediated caspase-1 activation or GSDMD cleavage, which leads to pyroptosis [103,104,107]. In addition, it was shown that active caspase-8 could directly process recombinant GSDMD, which is sufficient to induce pyroptosis [104]. In agreement with this notion, an independent study showed caspase-8 cleaves GSDMD at D276, the same site used by caspase-1 [108]. In addition, caspase-8 was shown to induce GSDMD-mediated cell death in epithelial cells in the context of the lack of both apoptosis and necroptosis [109]. Overall, these results indicate that caspase-8 is another effector for the cleavage of GSDMD and for the induction of pyroptosis.

## 4. Other Inflammation-Related Processes in which Caspase-8 Might Be Involved

### 4.1. Autophagy

Autophagy is an intracellular self-degradation system that is responsible for the elimination of unwanted or potentially dangerous intracellular materials, such as protein aggregates or old/damaged mitochondria [110]. Autophagy is known to provide nutrients to the cell during fasting or other forms of stress.

In addition, autophagy also has been implicated in a number of biological processes, including development, aging, cancer, neurodegenerative diseases, and inflammation [111,112,113,114]. Autophagy is regulated by numerous proteins, the products of autophagy-related genes (Atg), such as ULK, ATG5, Beclin 1, and ATG7, and occurs even under normal physiological conditions. However, the autophagic process is enhanced by numerous factors, including nutrient deprivation and pharmacological inhibitors of the mammalian target of rapamycin (mTOR) [110,115].

Autophagy can regulate immune responses by influencing the development and survival of various inflammatory cells and the production of cytokines from them [116]. Related to inflammasome activation or IL-1β production, autophagy functions as a negative regulator [117,118,119]. Therefore, the disruption of autophagy in macrophages or dendritic cells through genetic deletion or the knockdown of the essential components of autophagy, such as Atg7, Atg16L1 showed enhanced the processing and secretion of IL-1β and IL-18 in response to TLR stimulation [117,118]. In human peripheral blood mononuclear cells (PBMCs), the inhibition of autophagy by 3-Methyladenine enhances IL-1β secretion in response to TLR stimulation [119].

Conversely, the induction of autophagy with rapamycin attenuated the release of IL-1β in response to LPS with ATP or alum [120].

Caspase-8 has been shown to regulate the autophagy process by targeting autophagic components, such as ATG3, ATG5 and Beclin-1 [121,122,123,124]. In proliferating T cells, caspase-8 regulated the excessive autophagy through the cleavage of RIPK1, which is associated with ATG16L and ATG5 [121].

Moreover, the inhibition of autophagy by caspase-8 leads to the enhancement of the cytotoxicity of melanoma in the treatment of TRAIL under arginine deprivation [122]. The same study showed that the activated caspase-8 induced by TRAIL cleaves Beclin-1 and Atg5 [122], and the truncated C-terminal Beclin-1 fragment localizes at the mitochondria, which promotes apoptosis [123]. A regulatory component of the autophagosome, Atg3 is another substrate of caspase-8 [124]. During TNF or TRAIL-induced apoptotic cell death, caspase-8-mediated Atg3 cleavage occurs, and the overexpression of non-cleavable Atg3 shows autophagic activity upon death receptor stimulation [125].

Together, these results indicate that caspase-8 plays a role in the inhibition of autophagy during apoptosis, which might contribute to accelerating the removal of unwanted cells by non-immunogenic cell death.

### 4.2. Ferroptosis

Ferroptosis is a new type of immunogenic cell death and is a kind of regulated necrosis[126,127,128]. Ferroptosis occurs with iron dependence and is usually accompanied by a large amount of iron accumulation and lipid peroxidation during the cell death process [127]. Although ferroptosis is distinct from other types of cell death, such as apoptosis, necroptosis, and autophagy in biochemical and morphological features, recent studies have suggested an interplay between ferroptosis and other types of cell death, including extrinsic apoptosis and necroptosis, in which caspase-8 is associated [129,130,131]. In human pancreatic cancer and human colorectal cancer cells, ferroptotic agents, such as erastin and artesunate, sensitize the cells to TRAIL-induced apoptosis [130]. The deficiency of acyl-CoA synthetase long-chain family member 4(ACSL4), a marker of sensitivity to ferroptosis, results in an increase in MLKL; conversely, a lack of MLKL increases the ferroptosis-sensitivity of NIH3T3 cells, suggesting that ferroptosis and necroptosis have compensatory roles to each other, in that resistance to one pathway sensitizes the cells to death by the other pathway [131].

However, very few studies have been performed on this topic, and there is no explicit proof to explain the role of caspase-8 in ferroptosis. Further studies on this issue are required to better understand if caspase-8 plays a role in ferroptosis.

## 5. Conclusions

In recent decades, several interesting studies have unveiled the multifaceted nature of caspase-8 in inflammation processes. As a result, it has become evident that caspase-8 functions as a proinflammatory and anti-inflammatory regulator, depending on the cellular context and the cell type (Table 1). In particular, caspase-8 is multifunctional in inflammasome activation. It can be a negative regulator for MLKL-mediated inflammasome activation, whereas it contributes to inflammasome activation and IL-1β release. Caspase-8 can also directly process pro-IL-1β or GSDMD and induce pyroptosis and the release of bioactive IL-1β.

These accumulated data have expanded the functions of caspase-8, from the initial concept of an initiator caspase in extrinsic cell death signaling to a regulator in cell survival and a variety of inflammatory processes.

## Figures and Tables

**Table 1:** Caspase-8–mediated pro-and anti-inflammatory functions.

Roles	Comments	References
**Anti-inflammation**		
Inhibition of necroptosis	Caspase-8 limits necroptosis by cleavage of RIPK1, RIPK3 and CYLD.	[10,11,12]
	Caspase-8 maintains the gut barrier by preventing necroptosis of infected cells	[93,109,132]
	Caspase-8 deficiency or lack of its activity promotes TNF-induced necroptosis.	[38,39,40]
Inhibition of inflammasome	The deficiency of caspase-8 causes LPS-induced inflammasome activation mediated by RIPK1-RIPK3-MLKL	[54,55]
Inhibition of inflammatory signaling	The expression of non-cleavable RIPK1 by caspase-8 in human results in auto-inflammatory disease	[62,63]
	The deficiency of caspase-8 can facilitate the activation of proinflammatory genes in a necroptosis independent manner	[61]
	Caspase-8 suppresses the cytosolic RNA sensor RIG-I-induced proinflammatory gene expression through limiting RIPK1 function.	[60]
	Caspase-8 can suppress an inflammatory pathway downstream of cytosolic innate DNA receptor in cultured keratinocytes	[42]
**Proinflammation**		
Inflammasome activation	Internalized bacteria induce caspase-8-mediated NLRP3 inflammasome activation	[89]
	Caspase-8 is required for pathogens (Yersinia, *Salmonella*), YopJ, cytoplasmic dsRNA, or apolipoprotein C3-induced NLRP3 inflammasome activation	[77,107,133,134]
	LPS triggering in cIAP KO cells induces caspase-8 promoted NLRP3 activation	[91]
IL-1β processing	TLR stimulation under stress conditions (cIAP inhibition, a chemotherapeutic drug, ER stress) leads to caspase-8-dependent IL-1β processing	[92,101,102]
	Fas or Dectin-1 activation induces caspase-8-mediated IL-1β processing	[95,135]
	In the EAE mouse model, caspase-8 processes IL-1β by forming IRAKM-casp8-ASC complex in microglia	[98]
Pyroptosis induction	Catalytic dead-caspase-8 causes pyroptosis -dependent perinatal mouse death.	[58,136]
	Yersinia infection or TAK1 blocking trigger caspase-8-mediated GSDMD processing and pyroptosis induction	[103,104,137]
NF-κB activation, cytokine production	Caspase-8 is required for NF-κB activation and cytokine production by antigen receptor, TLR triggering or TRAIL stimulation.	[55,66,70,78,81]
